# Costs of dengue in three French territories of the Americas: an analysis of the hospital medical information system (PMSI) database

**DOI:** 10.1007/s10198-015-0694-9

**Published:** 2015-05-12

**Authors:** M. Uhart, C. Blein, M. L’Azou, L. Thomas, L. Durand

**Affiliations:** Global Health Sanofi Pasteur, Lyon, 69007 France; Health Economist, HEVA, 186 Avenue Thiers, 69465 Lyon Cedex 06, France; Global Epidemiologist, Sanofi Pasteur, Lyon 69007, France; Former head of the emergency department, Centre Hospitalier Universitaire, 97200 Fort-De-France, Martinique; Global Health Economist, Sanofi Pasteur, Lyon 69007, France

**Keywords:** Dengue, Hospitalisation cost, Economic burden, French Guiana, Guadeloupe and Martinique, I1 Health/I120 Health production

## Abstract

**Background:**

Dengue is a major emerging public health concern in tropical and subtropical countries. Severe dengue can lead to hospitalisation and death. This study was performed to assess the economic burden of hospitalisations for dengue from 2007 to 2011 in three French territories of the Americas where dengue is endemic (French Guiana, Martinique and Guadeloupe).

**Methods:**

Data on dengue-associated hospitalisations were extracted from the French national hospital administrative database, Programme de Médicalisation des Systèmes dʼInformation (PMSI). The numbers of stays and the corresponding number of hospitalised patients were determined using disease-specific ICD-10 codes. Associated hospital costs were estimated from the payer perspective, using French official tariffs.

**Results:**

Overall, 4183 patients (mean age 32 years; 51 % male) were hospitalised for dengue, corresponding to 4574 hospital stays. In nearly all hospital stays (98 %; 4471), the illness was medically managed and the mean length of stay was 4.3 days. The mean cost per stay was €2522, corresponding to a total hospital cost of €11.5 million over the 5 years assessed. The majority of hospitalisations (80 % of patients) and associated costs (75 % of total hospital costs) were incurred during two epidemics.

**Conclusion:**

Severe dengue is associated with significant hospital costs that escalate during outbreaks.

## Introduction

Dengue is an infectious disease caused by dengue viruses, transmitted to humans by mosquitoes (*Aedes aegypti* and *Aedes albopictus)* [1, 2]. Infection with dengue may be asymptomatic in some cases, or may manifest as a range of symptoms from self-limited dengue fever lasting 2 to 7 days to the more severe life-threatening dengue haemorrhagic fever with shock syndrome including severe bleeding and/or severe organ impairment, and death. Severe dengue is a medical emergency requiring immediate hospital management including intravenous fluid replacement, oxygen administration and vital signs surveillance. There is no specific antiviral treatment for dengue: current practice focuses on the alleviation of symptoms. Most patients recover fully from dengue, but some may experience fatigue and depression lasting several weeks [3, 4].

At present, prevention or reduction of dengue virus transmission depends entirely on the control of the mosquito vectors or interruption of human-vector contact. These methods alone have thus far demonstrated to be insufficient to control the disease [1]. In 2009, the World Health Organization (WHO) estimated that approximately 2.5 billion people lived within the hundred inter-tropical countries where the disease is endemic. With a worldwide incidence estimated at 50–100 million cases per year, dengue is the most rapidly spreading viral disease, with incidence rates having increased 30-fold in the last 50 years [2]. More recently, Bhatt et al. [5] using cartographic modelling approaches with data from a combination of published literature and online resources for the period 1960 to 2012, estimated that there may be approximately 390 million dengue infections (symptomatic and asymptomatic) per year, of which 96 million (67–136) experienced manifest disease (of any severity). Severe dengue causes an estimated 500,000 hospitalisations every year [2] and approximately 20,000 deaths annually [6].

Dengue is also an increasing public health concern in the Americas where there has been a 4.6-fold increase in the number of cases over the last three decades (from approximately 1 million cases during the 1980s to 4.7 million during 2000–2007) [7]. With an ever increasing number of cases, the economic burden inflicted on those populations in endemic areas is substantial [8–10]. In the Americas, the economic burden associated with dengue was estimated to be US$2.1 billion [11]. French Guiana, Martinique and Guadeloupe (three French territories in the Americas) are dengue endemic areas with cyclical epidemics every 3–5 years. The availability of an exhaustive official French hospital database represents a unique opportunity to assess the burden of dengue in these territories where the population is approximately 1 million (around 400,000 in Guadeloupe, 380,000 in Martinique and 250,000 in French Guiana) [12]. To date, only one publication has reported costs associated with dengue in these three territories, but the costs reported were based on extrapolation from other countries in the Americas [11]. The objective of the present study was to assess the cost associated with hospitalisation for dengue in French Guiana, Martinique and Guadeloupe from 2007 to 2011.

## Methods

### Data sources

The French Medical Information System (Programme de Médicalisation des Systèmes dʼInformation, PMSI) is an exhaustive medico-administrative hospital discharge database that covers all public and private hospitals in France as well as those in the French Territories [13–15]. Diagnoses identified during admission are coded using the International Classification of Diseases, 10th revision (ICD-10) by the physician. PMSI includes a compilation of standard discharge summaries (“Résumé Standard de Sortie”, RSS) for every admission. Anonymised data (“Résumé Standardisé Anonymisé”, RSA) with limited socio-demographic information (gender, age, residence code) and medical information on the main diagnosis that led to hospital admission, the nature of treatments received and examinations carried out, underlying comorbidities and possible complications, are made available for epidemiologic studies. Each patientʼs stay is classified by Diagnosis Related Group (DRG) (Groupe Homogène de Séjours) according to information documented by the physician.

The economic burden of hospitalised cases would be expected to be well documented within the PMSI database because since the introduction of a DRG-based prospective payment system (the “Tarification à l’Activité”) in 2005, the PMSI database has been used as the basis for the funding of services in all hospitals, with each hospital receiving DRG-based payments according to the national tariff. Thus, data extracted from this database is exhaustive (all public and private hospitals are included and no sampling is done) and of high quality, with limited coding errors. In addition, the FICHCOMP (“FiICHier COMPlémentaire”) database contains a restricted list of “expensive drugs” that are fully reimbursed, and has been available since 2008 for public hospitals only.

### Data collection of dengue stays

All hospital stays in the French territories of French Guiana, Martinique and Guadeloupe from 2007 to 2011 (data from more recent years were not available at the time of analysis) with a primary or associated dengue-specific code were selected from the PMSI database using the ICD-10 codes A90* or A91* [“Dengue fever (classical dengue)” or “Dengue haemorrhagic fever (severe dengue)”, respectively]. In this first data collection, we gathered hospital stays for confirmed dengue or suspected dengue. In a second step we assessed a medical interpretation in order to select the confirmed dengue cases completed by PMSI guidelines. In this second step, we used the PMSI guidelines to firstly select confirmed dengue cases based on ICD-10 diagnosis code A90* or A91* in position of primary diagnosis. When the ICD-10 diagnosis code A90* or A91* is observed in position of associated diagnosis, the medical interpretation is absolutely necessary. Based on the medical interpretation of PMSI database stays and particularly the primary diagnosis, the dengue was confirmed or not.

A conservative approach was used by selecting only those hospital stays that had a primary diagnosis of dengue or had a direct link with dengue. One of the authors (LT) manually assessed all hospital stay cases that had an associated link to dengue (i.e. where dengue was a secondary diagnosis) to exclude those where dengue was considered doubtful.

For patients hospitalised for dengue, gender, age and comorbidities, type of management received (medical, surgical or exploratory), type of stay (conventional inpatient stays or short outpatient stays), and length of stay, as well as whether stays occurred partly, or completely, in the emergency or an intensive care unit, and occurrence of related deaths were collected. Conventional inpatient stays include day hospitalisations defined as an admission of 2 or more days’ duration, whereas short stays include day hospitalisations. Since patients may have several hospital stays during the year, the overall number hospitalised at least once over a given period could be obtained by linking all hospital stays with anonymised patient identification numbers based on the patient’s social security number, date of birth and gender.

### Economic burden of dengue hospitalisation

Costs were estimated from the social security payer perspective, i.e. Ministry of Health public fund. Ambulatory costs and indirect costs related to productivity loss were not considered in the present study. Hospital-associated costs were calculated using official DRG tariffs and “expensive drug” tariffs for each year considered. DRG tariffs represent the willingness-to-pay by the national health insurance and not the hospital cost production. DRG tariffs include medical and related procedures, nursing care, treatments (except specific expensive drugs), drugs used, food and accommodation, and investment costs for hospitalised patients. Additional cost per day of hospitalisation in emergency or an intensive care unit was added to DRG tariffs, when appropriate. For private hospitals, physician’s fees were also added to the DRG tariffs; physicians are reimbursed on a fee-for-service basis (source: ENCC 2010). Costs are presented as mean cost per stay, mean cost per patient and total cost per year for the three French territories in the Americas. All costs are presented in euros (€).

## Results

**Fig. 1:**
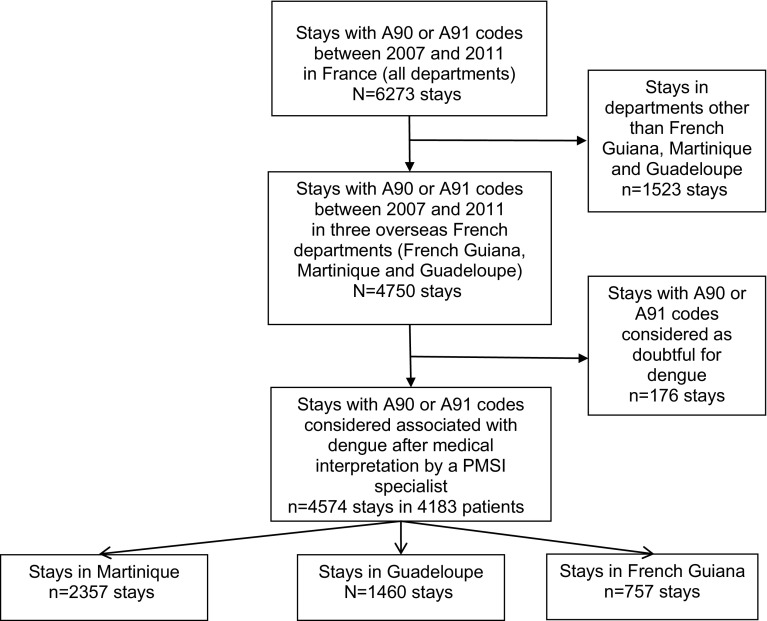
Flow-chart of stays selected for the analysis. A90* and A91* correspond to ICD-10 codes for dengue fever (classical dengue) and dengue haemorrhagic fever, respectively. *A given patient may be hospitalised in more than one department over the period

**Fig. 2:**
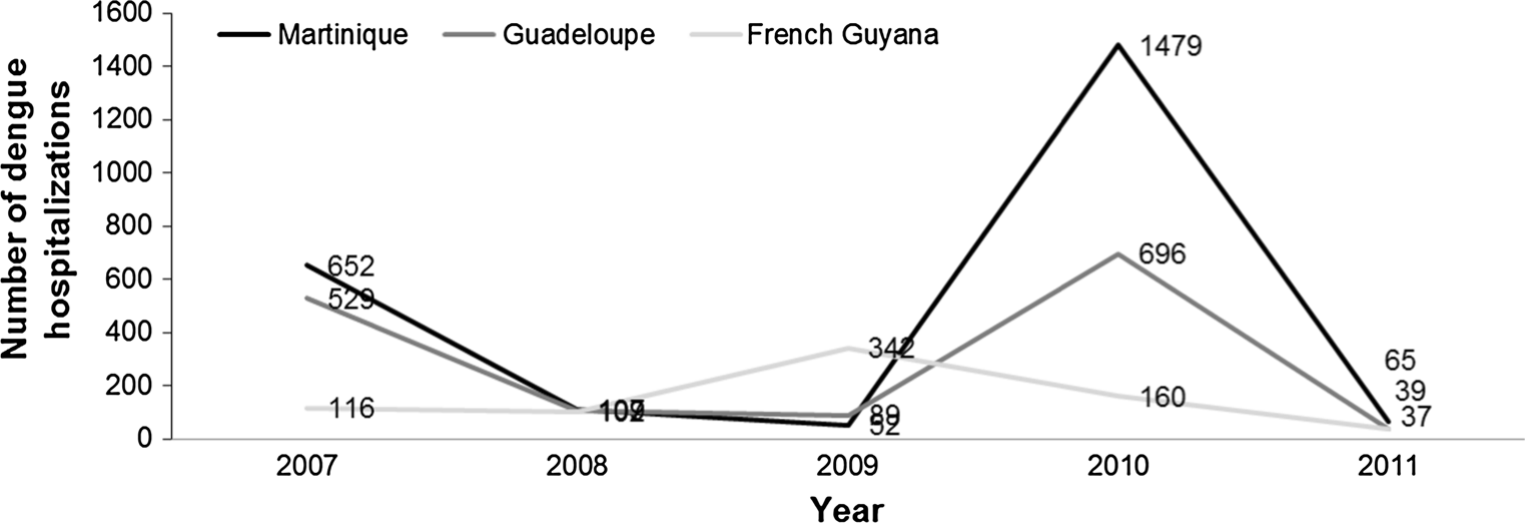
Annual number of hospitalisations for dengue from 2007 to 2011 in each of the three French territories*. *A given patient may be hospitalised in more than one department over the period

A total number of 6273 hospital stays for dengue were identified in the PMSI database between 2007 and 2011, including 4750 stays (76 %) that occurred in French Guiana, Martinique or Guadeloupe (Fig. 1). Of the hospital stays in the three French territories, 176 (4 %) were considered to be doubtful for dengue and were excluded. The remaining 4574 hospital stays constituted the base case of the present analysis. Overall, 4183 patients were hospitalised for dengue during the study period: 622 (15 %) patients in French Guiana; 2231 (53 %) in Martinique; and 1354 (32 %) in Guadeloupe. A patient may have several dengue hospitalisations in different territories, resulting in a non-arithmetic total of patients over all the three territories. Three peaks in the overall number of hospital stays were observed during the study period (Fig. 2): 28 % of hospital stays occurred in 2007, 11 % in 2009 and 51 % in 2010. Among the total number of patients hospitalised in the three French territories during the study period, patients hospitalised for dengue represented 0.81 % (*n* = 1184) of patients in 2007, 0.17 % (*n* = 237) in 2008, 0.29 % (*n* = 445) in 2009, 1.35 %(*n* = 2204) in 2010 and 0.08 % (*n* = 129) in 2011.

**Fig. 3:**
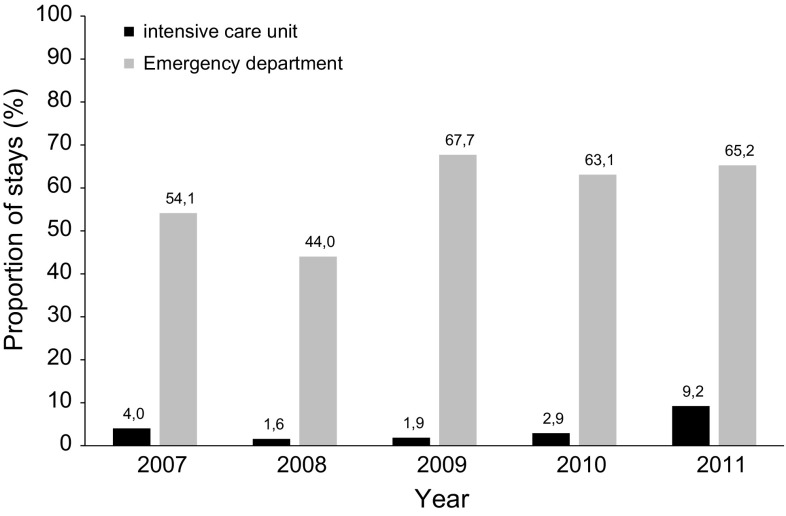
Percentage of stays where patients were admitted via the emergency or intensive care unit by year

**Fig. 4:**
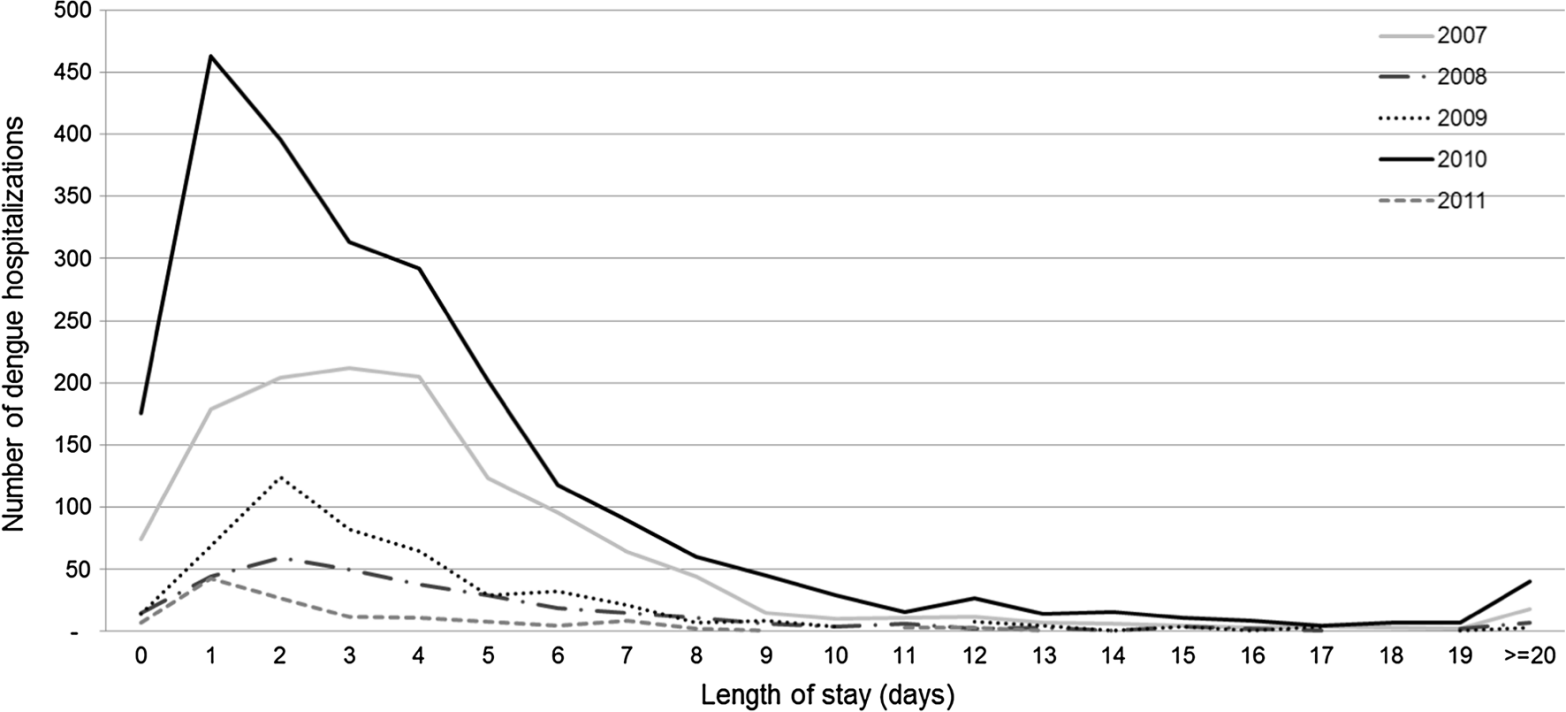
Distribution of the length of stay by year

Males represented 51 % (*n* = 2146) of cases, and the overall mean age was 32 (SD 23) years. Children and young adults (aged younger than 19 years) accounted for 43 % (1962) of stays. More than half of stays (55 %; 2533) had no underlying comorbidity. Over the 2021 stays occurred with at least one comorbidity, sickle-cell disease was recorded in 6 % (126) of stays. Most hospital stays [60 % (2734); range 44 % (140/318) for the year 2008 to 68 % (327/483)] for the year 2009 were initially admitted via the emergency department (Fig. 3), representing 2734 stays. Only 147 (3 %) hospital stays were initially admitted via the intensive care unit. Forty (1 %) patients died during their stay. Conventional inpatient dengue stays accounted for 84 % (3839 stays) of all hospital stays. The median length of dengue stay was 3 days (range from 0 to more than 20 days) and the mean length of stay was 4.3 days (SD 7). The length of dengue stay was less than 5 days in 69 % (3174 stays) of stays, and was similar year on year (Fig. 4).

In nearly 4471 dengue stays (98 %), the illness was medically managed, with surgical intervention recorded in <1 % (26) of stays.

### Economic burden of dengue hospitalisation

Table 1 summarises the mean cost (based on national DRG tariffs) per stay, mean annual cost per patient and the total annual cost of hospitalisation for dengue for the three French territories. The mean cost per stay during the study was estimated at €2522 (SD €3707). The total hospitalisation cost amounted to €11.5 million, of which, 27, 10 and 52 % of these costs were incurred in 2007, 2009 and 2010, respectively. The total cost of expensive drugs used in treatment between 2008 and 2011 was €143,453, of which 94 % (€135,144) of the cost was incurred in 2010. In 2010, three drugs counted for 92 % of the expensive drug costs for that year: €56,334 (42 %) for the use of Benefix^®^ (coagulation factor IX recombinant), €43,552 (32 %) for Tegeline^®^ (human immunoglobulin) and €24,537 (18 %) for Novoseven^®^ (coagulation factor VIIa recombinant). The mean cost per patient was estimated at €2758 (SD € 9605), range €2588 (SD €5040) in 2007 to €4138 (SD € 11,920) in 2011.

**Table 1:** Cost per stay, annual cost per patient and total annual costs from the social security perspective

	Number of stays	Stays admitted via the intensive care unit* (%)	Mean (±SD) cost per stay (€)	Mean (±SD) annual cost per patient (€)	Total annual cost (€)
2007	1297	4	2362 ± 2454	2588 ± 5040	3,064,199
2008	318	1.6	2765 ± 2682	3710 ± 14,649	879,370
2009	483	1.9	2312 ± 2074	2509 ± 4080	1,116,874
2010	2335	2.9	2545 ± 3707	2696 ± 4215	5,942,788
2011	141	9.2	3786 ± 11,441	4138 ± 11,920	533,866
2007–2011	4574	3.2	2522 ± 3707	2758 ± 9605	11,537,098

* With financial supplement

## Discussion

### Main findings

The present study was designed to estimate the economic burden of hospitalisation related to dengue infections in three French territories of the Americas where dengue is endemic, between 2007 and 2011. The total hospitalisation cost reached €11.5 million over the 5-year period.

### What is already known on this topic

In terms of the average cost per stay, our results appear to be consistent with those presented by Shepard et al. [11] who estimated these at US$3460, US$3430 and US$4052 per hospitalised dengue case in 2010 (equivalent to €2494, €2473 and €2921 assuming US$1 = €0.72, 2014 exchange rate) in French Guiana, Martinique and Guadeloupe, respectively, versus an average of €2522 across the three territories in the present study.

### What this study adds

We observed that the annual number of hospital stays for dengue in Martinique and Guadeloupe appeared to mirror each other over the study period, with peaks in stays observed during 2007 and 2010. This may be explained by the relative proximity of these territories to each other and to the dengue epidemics observed in those years in these two territories. In French Guiana, the annual number of hospital stays peaked in 2009; again consistent with the occurrence of a dengue epidemic in that region during that year. Overall, the hospitalisation costs incurred during these dengue epidemics accounted for about 90 % of the overall costs over the study period.

The present study underlines the central role of the emergency department in the management of dengue. Overall, 60 % of hospitalisations for dengue between 2007 and 2011 were initially admitted via the emergency department. Emergency departments allow both triage of patients who do not require hospitalisation and for those who do in severe cases. Unfortunately, the PMSI database does not record information on patients who attend the emergency department but who do not require subsequent hospitalisation. It would be interesting to analyse the number of patients who attend the emergency department for dengue but who do not require subsequent hospitalisation and their related associated costs, especially during an epidemic. Epidemics represent significant challenges for health-care providers with regard to delivering care to a large number of patients in a short period of time, as well as identifying those at risk of developing severe dengue requiring hospitalisation [1]. For instance, in Martinique in the 2010 outbreak, it was observed that dengue represented more than 10 % of the total emergency admissions, while this usually represents less than 1 %, which could lead to a possible saturation of the emergency department [16].

Although outbreaks were associated with qualitative changes in the management and in the profile of hospitalised patients, this was not reflected in the cost per dengue case. For instance, the use of expensive drugs was predominant during the 2010 outbreak, which accounted for 94 % of the overall costs for these expenses. In addition, the proportion of patients hospitalised in intensive care units was also increased during the 2007 and 2010 epidemics. Nonetheless, these variations in costs had a negligible impact on the average annual costs per case (Table 1). However, as the PMSI database is based on the collection of coding added by various health professionals, it is possible that coding bias may have contributed to this observation. At the opposite end, the cost per stay was highest in 2011, a dengue non-epidemic year. The higher costs per stay observed in 2011 may be attributed, in part, to the higher rate of admission to the intensive care unit compared to other years (9.2 % with* n* = 13 stays versus an over mean at 3.2 % with* n* = 147 stays). This observation may simply reflect “classic disease management” during a non-epidemic period following an epidemic, where the few patients with disease receive intensive treatment and monitoring—whereas during epidemics, in order to avoid intensive care unit saturation, a more rigorous selection of severe cases with complications may occur.

## Limitations of this study

In addition to the direct costs associated with dengue, which escalate during epidemics, dengue prevention measures such as vector control through use of larvicides and fumigation as well as education, media, and community campaigns, incur additional costs that are not captured within the PMSI database. The additional costs incurred for prevention measures have been reported for both the 2007 epidemic in Guadeloupe and the 2010 epidemic in Martinique. During the epidemic of 2010 in Martinique, €60,000 and €120,000 were spent on insecticides and communication, respectively, and extra funding of €100,000, given by the government, was used mainly for educational campaigns [16]. In Guadeloupe, the cost of a communication campaign for the 2007 epidemic was reported as €130,424, of which, €98,542 was paid by the national government with the remainder paid by various external partners, private organisations or municipalities [17]. Of note, the cost of the communication campaign represented 20 % of the global governmental budget for communication in Guadeloupe for 2007.

In order to estimate the global economic burden of dengue, costs outside the hospital setting associated to ambulatory care (pre- and post-hospitalisation costs incurred by a dengue hospital case, and the ambulatory costs incurred by a dengue ambulatory case) and costs related to productivity losses would need to be assessed. Indeed, the majority of dengue infections are treated in an ambulatory setting. In the Americas [11] ambulatory cases (with ambulatory care only) accounted for 73 % of the overall total direct costs for the management of dengue, but with high variability between regions. Taking into account this breakdown of costs, the total direct costs associated with dengue in the three French territories could reach €42.6 million (€11.5 million for hospitalised cases and €31.1 million for ambulatory cases, assuming that ambulatory cases represent 73 % of the total). This total direct cost could increase by estimating also the ambulatory care pre- and post- hospitalisation. Indirect costs associated with loss of work or school absenteeism for the patient or the caregiver are also considerable. An estimated 60 % of the total costs associated with dengue in the Americas corresponded to indirect costs, mostly related to productivity losses [11]. In addition, recent evidence indicates that dengue causes a substantial reduction in quality of life during infection that lasts longer than the duration of fever [3, 4, 19–21]. Chronic fatigue may negatively impact on daily activities and ability to work for up to 2 years after infection [3, 4], with associated productivity losses that may be substantial.

## Conclusions

The present analysis is a first step in the estimation of the global economic burden of dengue in French Guiana, Martinique and Guadeloupe, three French territories of the Americas where dengue is endemic. To present the complete economic burden, ambulatory costs would also need to be collected as well as indirect costs associated with dengue infections. However, the costs of hospitalisation reported in our study already highlight the economic impact of dengue, especially during epidemics. Further studies analysing the cost of dengue are needed considering that several vaccine candidates [21] and other prevention and control technologies are currently under development [22–25]. These studies will help determine the most efficient strategy in the management of dengue.
